# Great diverse rhizobial community nodulating *Astragalus mongholicus* in the northeastern region of China

**DOI:** 10.3389/fmicb.2024.1507637

**Published:** 2024-12-13

**Authors:** Mengzhe Gao, Xiaoxia Yuan, Zhaojun Ji, Bingjie Yang, Hua Li, Bo Zhang

**Affiliations:** ^1^College of Life Science and Food Engineering, Inner Mongolia Minzu University, Tongliao, China; ^2^Inner Mongolia Autonomous Region Engineering Technology Research Center for Prevention and Control of Pathogenic Bacteria in Milk, Tongliao, China; ^3^Key Laboratory of Mongolian Medicine Research and Development Engineering, Ministry of Education, Tongliao, China

**Keywords:** *Astragalus mongholicus* Bunge, rhizobial strains, diversity, gene flow, genetic differentiation

## Abstract

**Introduction:**

*Astragalus mongholicus* Bunge is an important medicinal legume species widely cultivated in northeastern China (NEC) and northwestern China (NWC) and can establish a symbiotic relationship with nitrogen-fixing rhizobial strains. However, there are limited reports comparing the genetic diversity, differentiation, and gene flow of rhizobial strains associated with this plant in different geographic regions.

**Methods:**

We used multilocus sequence analysis (MLSA) to investigate the phylogeny and genetic diversity of rhizobia and to estimate their intra- and inter-regional gene flow and genetic differentiation based on the analysis of concatenated core genes (*recA*, *atpD*, and *glnII*) and the critical symbiotic gene *nodC*.

**Results:**

We isolated eight known and three novel genospecies representing four genera, among which *Rhizobium yanglingense* was the most predominant microsymbiont. Phylogenetic analysis revealed a highly diverse rhizobial community nodulating *Astragalus mongholicus* in NEC, consisting of the four genera *Rhizobium*, *Bradyrhizobium*, *Sinorhizobium*, and *Mesorhizobium*. This community differed markedly from the rhizobial community found in NWC. Various rhizobial genospecies with different symbiotic gene *nodC* sequences were capable of nodulating *A. mongholicus* in NEC. Therefore, *A. mongholicus* exhibits promiscuity in its association with symbionts in the natural environment, showing no strong preference for either the species-defining core genes or the symbiotic genes of rhizobia. We also found that the Glyco_tranf_GTA_type superfamily (Glycosyltransferase family A) is the most highly conserved and essential domain in the NodC protein, which is encoded by the symbiotic *nodC* gene, across nodulating rhizobia. In addition, we found independent genetic differentiation among rhizobial communities geographically, and the frequency of gene flow among microsymbionts between NEC and NWC was low. We speculate that the formation of the highly diverse rhizobial community in NEC resulted from the independent evolution of each ancestral lineage. This diversity likely arose from intraregional genetic differentiation driven by mutations rather than recombination.

**Conclusion:**

Ecogeographical isolation between NEC and NWC restricted inter-regional genetic drift and gene flow. Therefore, intraregional genetic differentiation is the major evolutionary force underlying the genetic diversity of rhizobia.

## 1 Introduction

The plant genus *Astragalus* L. (Fabaceae) includes 2,704 species that are recognized as “accepted” in the Plant List (TPL), a dataset that serves as the taxonomic backbone of the World Flora Online.^1^ It is the largest genus in the Fabaceae family, with a wide distribution worldwide (Chen et al., 2015). Many *Astragalus* species form nitrogen-fixing symbioses with rhizobia (Wei et al., 2003; Tian et al., 2021) that can fix atmospheric nitrogen and convert it to ammonia, which is then absorbed by their host plants, thereby reducing the need for nitrogen fertilizers (Zhou et al., 2024). More than 20 rhizobia belonging to *Rhizobium*, *Mesorhizobium*, *Sinorhizobium*, *Bradyrhizobium*, and non-nodulating *Agrobacterium* have been isolated from the root nodules of 14 *Astragalu*s species distributed across 12 Chinese provinces or autonomous regions, including Xinjiang, Inner Mongolia, Liaoning, Beijing, Shandong, Gansu, and Yunnan. These provinces encompass five different ecological regions, namely temperate-arid, semi-arid agricultural, semi-arid, Loess Plateau, and subtropical regions (Gao et al., 2001; Zhao et al., 2008; Chen et al., 2015; Yan et al., 2016; Zhang et al., 2020b). The genetic diversity and biogeographic distribution of rhizobia nodulating *Astragalus* are primarily influenced by soil contents of available phosphorus and potassium, as well as total salts, pH, and soil fertility (Wei et al., 2008; Yan et al., 2016). *Mesorhizobium jarvisii* is the most widespread and predominant species associated with *A. sinicus* in southwestern China (Zhang et al., 2020b), where the majority (53%) of isolates are non-nodulating *Agrobacterium* sp. Meanwhile, in the arid region of northwestern China (NWC) (Chen et al., 2015), symbiotic *Mesorhizobium* species comprise 41% of the rhizobial isolates. *M. septentrionale* and *M. temperatum* are the predominant species associated with *Astragalus* in fields with nitrogen-poor and -rich soils, respectively (Yan et al., 2016). In the Kamchatka Peninsula, genera that have been isolated from nodules of *A. umbellatus* and *A. inopinatus* are *Mesorhizobium*, *Rhizobium*, *Bosea*, and *Bradyrhizobium* (Guro et al., 2024).

*A. mongholicus* is a perennial plant that serves as a primary medicinal herb in the arid and semi-arid regions of NEC and NWC, where it is mainly distributed (Fu et al., 2014; Wang et al., 2023; Li et al., 2024). It can form nodules with rhizobia. The microsymbionts associated with *A. mongholicus* in NWC (Shanxi, Gansu, and Ningxia provinces) include *M. temperatum*, *M. muleiense*, *M. septentrionale*, *M. ciceri*, *S. meliloti*, and *S. kummerowiae* (Yan et al., 2016). The microsymbionts associated with *A. mongholicus* in NEC have not been reported yet. The climate of this region is temperate semi-arid with windy and dry winters and springs, warm and rainy summers, and cool autumns. The mean annual temperature is 7°C; and 70% of the annual mean precipitation of 300 mm falls between July and September. The region’s black soil is characterized by its high content of humus (Yan and Liu, 2010; Xiulian et al., 2017). These characteristics differ from those in NWC, where the climate is arid and semi-arid temperate continental monsoon; the average annual precipitation of 358.2 mm occurs mainly from July to September; and its soil is sandy with a low humus content (Meng et al., 2015).

Systematic studies on the genetic diversity and biogeographic distribution of rhizobia associated with various legumes have revealed significant differences among populations in different ecoregions (Liu G. et al., 2021; Liu L. et al., 2021). Genetic differentiation, gene flow, and allelic substitution within and between rhizobial populations may arise from mutation and recombination, resulting in specific patterns of adaptation in response to environmental variation and host selection. For instance, the biogeography of populations of *M. muleiense*, one of the main chickpea-nodulating rhizobial symbionts in China, reveals a pattern of chromosomal differentiation among populations from the Ningxia, Xinjiang, and Gansu provinces (or autonomic regions) (Zhang et al., 2020a). *Caragana*-associated mesorhizobia have evolved divergently according to the interplay between environmental conditions and host plants (Ji et al., 2015). In contrast, gene exchange and recombination among *Bradyrhizobium* associated with *Kummerowia* have occurred more frequently among the genospecies isolated from exurban and urban areas, regardless of the geographical distribution (Ji et al., 2019). However, little is known about the genetic diversity, intraregional differentiation, and gene flow among rhizobial strains associated with *A. mongholicus* in NEC. Moreover, the differences between the evolutionary trajectories of these strains in NEC and NWC are also unclear.

To address this knowledge gap, we collected the root nodules of *A. mongholicus* at a plantation. The plantation maintains a variety of Chinese herbaceous plants such as *A. mongholicus*, *Glycyrrhiza uralensis*, *Sophora flavescens*, *Saposhnikovia divaricata*, and *Platycodon grandiflorus*. We used multilocus sequence analysis to investigate the phylogeny and genetic diversity of rhizobial isolates associated with *A. mongholicus*, and we systematically estimated the intra- and inter-regional differentiation and gene flow among these isolates based on the analysis of the concatenated core genes (*recA*, *atpD*, and *glnII*) and the key symbiotic gene *nodC* (encoding N-acetylglucosaminyltransferase, NodC).

## 2 Materials and methods

### 2.1 Nodule collection and rhizobia isolation

Nodules on the roots of *A. mongholicus* were collected from a herbaceous plantation named “Traditional Chinese and Mongolian Medicinal planting demonstration base for revitalization of technology” located in Naiman County, Inner Mongolia of NEC (42°34′45″N, 120°45′35″E). The nodules were carefully excised by hand from the roots of *A. mongholicus* 3 months after sowing at the flowering stage when the leaves were green and the plant had reached a height of approximately 20 cm. Nodules were placed into Eppendorf tubes and transported to our laboratory 2 h after collection. The nodules were then washed with 0.85% saline solution and then aseptically transferred to sterile containers. The washed nodules were treated with 95% ethanol for 30 s, followed by 2.4% sodium hypochlorite for 5 min, and rewashed seven times with sterile water. Each nodule was separately transferred to a sterile Eppendorf tube containing 50 μL of sterile saline and then crushed using sterile forceps. The whole mixture was streaked onto Yeast Mannitol Agar (YMA) medium and incubated for 72 h at 28°C. Single colonies were purified three times and then inoculated into YMA liquid medium (Kabdullayeva et al., 2020; Wekesa et al., 2021), followed by incubation at 28°C with shaking at 180 rpm for 72 h. The resulting bacterial suspension was mixed with 60% glycerol (1:1) and stored at −80°C (Cao et al., 2024; Das et al., 2024).

### 2.2 Characterization of the soil samples

Soil samples that were collected from the plantation in NEC (42°34′45″N and 120°45′35″E) were treated to determine their chemical properties. The contents of available N, P, K, and pH were analyzed in the Plant Nutrient and Resource Research Institution, Beijing Academy of Agriculture and Forestry Sciences (Beijing), following standard methods (Yan et al., 2014).

### 2.3 Isolate identification and nodulation test

DNA of each of the isolated strains was extracted using previously described methods (Terefework et al., 2001). All the isolates obtained in the present study were first identified by comparing the average nucleotide identity (ANI) of each isolate’s core *recA* gene with those of corresponding type strains. A total of 140 genotypes were identified as rhizobial strains (results not shown), and 34 rhizobial strains representing each genotype from NEC (area A) were selected for further study. In addition, we also selected 18 rhizobial strains associated with *A. mongholicus* in NWC (Supplementary Table S1). Specifically, we chose six strains from Shanxi (area B), five from Gansu (area C), and seven from Ningxia (area D), following the methods of Yan et al. (2016). In this study, we used 14 type strains (*R. yanglingense*^T^, *R. mongolense*^T^, *R. giardinii*^T^, *M. ciceri*^T^, *S. kummerowiae*^T^, *R. gallicum*^T^, *R. tropici*^T^, *S. fredii*^T^, *S. meliloti*^T^, *B. yuanmingense*^T^, *M. mediterraneum*^T^, *M. temperatum*^T^, *M. septentrionale*^T^, and *M. muleiense*^T^) as reference strains for the phylogenetic analysis of all rhizobial strains.

A total of 34 representative isolates were cultured separately in 5 mL of YMA broth with shaking up to the late exponential phase (about 48 h, OD_600_ ≈ 2.0). One milliliter of each culture was inoculated into *A. mongholicus* to test its nodulation capacity. The nodulation tests were carried out according to previously described methods (Yan et al., 2016). The nitrogen-fixing effectiveness of the nodules was evaluated based on the intensity of pinkness of the section of nodules and the intensity of greenness of the leaves.

### 2.4 Gene amplification and sequencing

We evaluated the genetic differentiation and gene flow among rhizobial strains isolated from both NEC and NWC based on the sequences of core (*recA*, *atpD*, and *glnII*) and symbiotic (*nodC*) genes. The details of the amplification protocols are presented in Supplementary Table S2 (Ji et al., 2015). The purified amplification products were commercially sequenced by using ABI 3730xl DNA Analyzer. All sequences were deposited in the National Center for Biotechnology Information (NCBI) database (Supplementary Table S5).

### 2.5 Phylogenetic analysis of core and symbiotic genes

The nucleotide sequences of these rhizobial isolates were aligned using the ClustalW program, and redundant sequences at both ends were removed (Aliliche et al., 2016). A neighbor-joining (NJ) phylogenetic tree of rhizobial isolates and 14 type strains was constructed using MEGA11 software. The analysis used a Kimura-2-parameter model based on three core (*recA*, *atpD*, and *glnII*) genes and one symbiotic (*nodC*) gene. The stability of the phylogenetic tree was estimated through bootstrap analysis in MEGA11 using 1,000 replicates (Huson and Bryant, 2006). The SplitsTree 4.0 program (Huson and Bryant, 2006) was used with 1,000 bootstraps to assess the degree of tree-like structure for alleles of each locus and concatenated sequences to reveal potentially incompatible signals in the evolutionary history of split phylogenetic networks.

### 2.6 Prediction of conserved domains in the NodC protein

The amino acid sequence of NodC was determined using MEGA 9.0 and was uploaded to the Batch CD-Search platform^2^ in the NCBI’s Conserved Domain Database (CDD), a freely available protein annotation tool. We used this tool to search for the conserved domains within the nucleotide sequence of *nodC*. The resulting predicted conserved domains were downloaded for visualization analysis using GraphPad Prism 9.5.1.

### 2.7 Nucleotide polymorphism and population structure

We used DnaSP (DNA sequence polymorphism) software to analyze nucleotide polymorphism by estimating the number of haplotypes (*h*), haplotype diversity (*Hd*), nucleotide diversity (*π*), *πN*/*πS* ratio (where *πS* indicates the number of synonymous site substitutions and *πN* indicates the number of non-synonymous site substitutions), average nucleotide genetic differentiation distance (*Dxy*), and gene flow index (*Nm*) (Nei and Gojobori, 1986) between NEC and NWC (Nei and Gojobori, 1986; Librado and Rozas, 2009). The minimal recombination events (*Rm*) within the populations were also estimated using DnaSP. We used STRUCTURE software (admixture-LOCPRIOR model, burn-in = 100,000, sampling iterations = 1,000,000) to measure the admixture levels of rhizobial genospecies distributed in all four areas (Didelot and Falush, 2007). We used ClonalFrame software to calculate two recombination rates (*r*/*m* and *ρ*/*θ*), where *r*/*m* is the relative impact of recombination compared to that of mutation in the genetic diversification of the ancestral lineage and *ρ*/*θ* is the relative occurrence frequency of recombination compared to that of mutation in the history of the ancestral lineage (Didelot and Falush, 2007).

## 3 Results

### 3.1 Phylogenetic analysis of the core genes and soil characteristics in NEC

**Figure 1:**
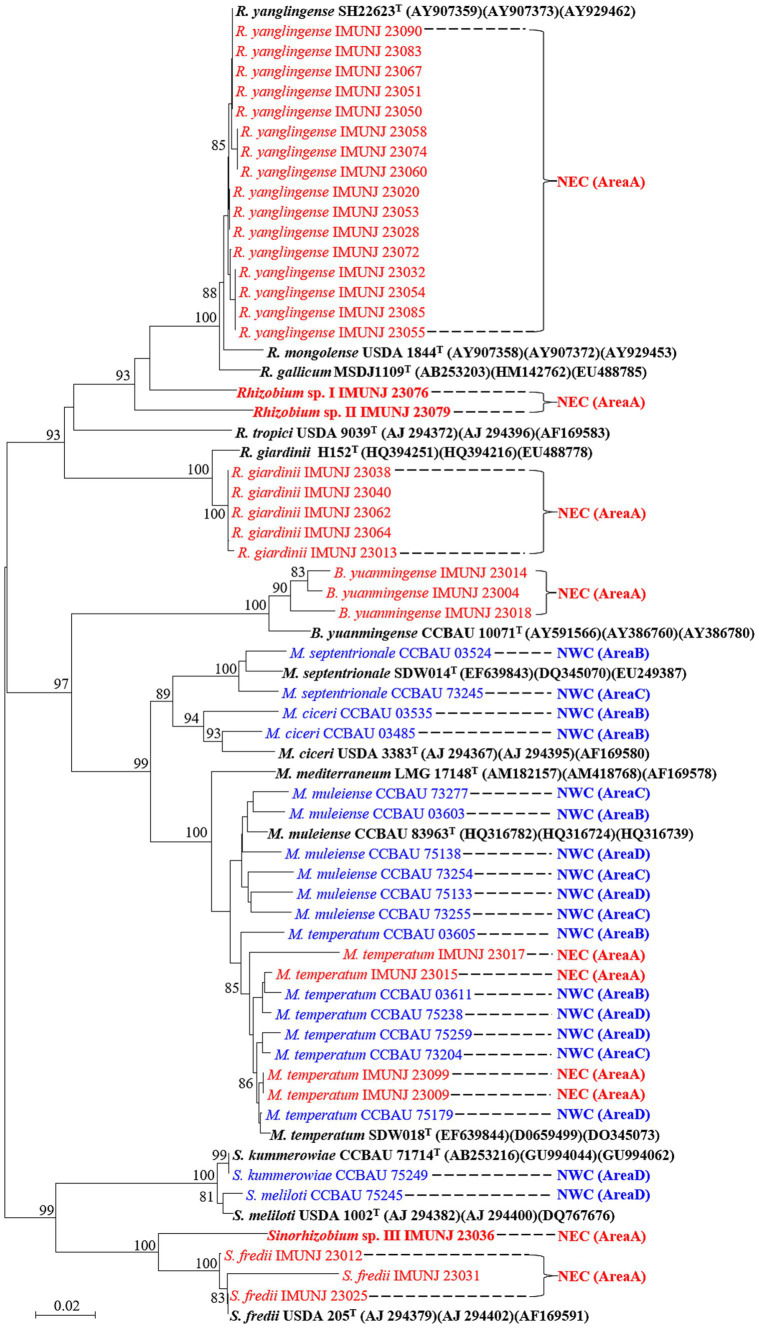
Neighbor-joining (NJ) tree of representative rhizobia associated with *A. mongholicus* from NEC (red) and NWC (blue) based on concatenated core genes (*atpD*, *glnII*, and *recA*). Bootstrap values greater than 70% are indicated at the branch points. The type strains (black bold fonts) are shown with GenBank numbers in parentheses. Novel genospecies (*Rhizobium* spp. I–II and *Sinorhizobium* sp. III) are shown in red bold typeface. The scale bar represents 1% nucleotide substitutions. The lengths of the *recA*, *atpD*, and *glnII* sequences are 440, 425, and 503 bp, respectively.

A total of 34 representative strains were selected for phylogenetic analysis based on the sequences of their *recA* gene, which is often used as a marker to determine taxonomy up to the species level (strain information shown in Supplementary Table S1). The NJ phylogenetic tree of three core genes (*glnII*, *atpD*, and *recA*) revealed five defined species, namely *Rhizobium yanglingense* (16 strains), *Rhizobium giardinii* (5 strains), *Bradyrhizobium yuanmingense* (3 strains), *Mesorhizobium temperatum* (4 strains), and *Sinorhizobium fredii* (3 strains), and three putative novel species, namely *Rhizobium* sp. I, *Rhizobium* sp. II, and *Sinorhizobium* sp. III, based on their ANI values. These rhizobial strains are encompassed by four genera: *Rhizobium*, *Mesorhizobium*, *Bradyrhizobium*, and *Sinorhizobium*. The most predominant microsymbiont in nodulated roots of *A. mongholicus* in NEC was *R. yanglingense*. An additional 18 rhizobial strains previously isolated from *A. mongholicus* planted in NWC (Yan et al., 2016) were also included in the phylogenetic analysis of the concatenated core genes (Figure 1). These rhizobial strains associated with *A. mongholicus* in NEC and NWC formed different groups in the phylogenetic tree, except for *Mesorhizobium temperatum* IMUNJ 23017, IMUNJ 23015, IMUNJ 23009, and IMUNJ 23099. The phylogenetic tree showed that *A. mongholicus* is capable of establishing a symbiotic relationship with *R. yanglingense*, *R. giardinii*, *S. fredii*, *B. yuanmingense*, and *M. temperatum* in NEC. However, it is associated with *M. muleiense*, *M. ciceri*, *M. septentrionale*, *S. kummerowiae*, *S. meliloti*, and *M. temperatum* in NWC.

The results of soil analysis showed that NEC has a slightly alkaline soil (pH 8.32), with organic carbon (OC), available potassium (AK), and available phosphorus (AP) contents of 8.73 g/kg, 108.63 mg/kg, and 10.41 mg/kg, respectively.

### 3.2 Phylogenetic analysis of the symbiotic gene *nodC*

**Figure 2:**
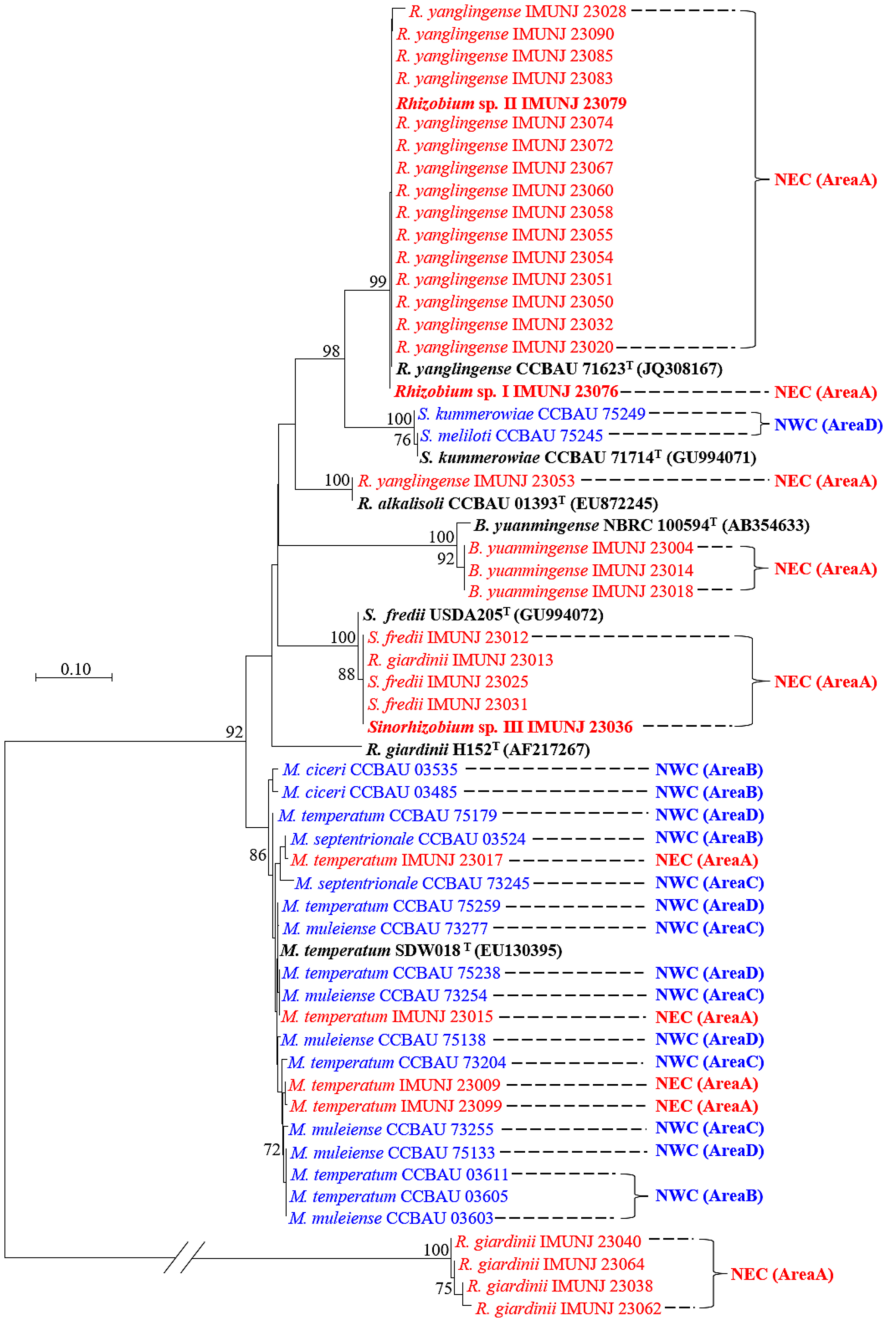
Neighbor-joining (NJ) tree of representative rhizobia associated with *A. mongholicus* from NEC (red) and NWC (blue) on the base of the symbiotic gene *nodC*. Bootstrap values greater than 70% are indicated at the branch points. The type strains (black bold fonts) are shown with GenBank numbers in parentheses. Novel genospecies (*Rhizobium* spp. I–II and *Sinorhizobium* sp. III) are shown in red bold typeface. The scale bar represents 1% nucleotide substitutions. The length of the *nodC* sequence is 429 bp.

The topological structures of the two phylogenetic trees constructed based on the sequences of the symbiotic gene *nodC* (Figure 2) and the concatenated core genes (Figure 1) using the NJ method were different. For instance, *Rhizobium* sp. I IMUNJ 23076 and *Rhizobium* sp. II IMUNJ 23079 shared the same branch as the type strain *R. yanglingense* CCBAU 71623. However, *R. yanglingense* IMUNJ 23053 shared the same branch as the type strain *R. alkalisoli* CCBAU 01393 (Figure 2). Figure 2 also shows an independent branch consisting of four strains including *R. giardinii* IMUNJ 23038, IMUNJ 23040, IMUNJ 23062, and IMUNJ 23064 at the bottom of the phylogenetic tree. However, *R. giardinii* IMUNJ 23013 is grouped with another branch next to other strains such as *S. fredii* IMUNJ 23012.

Analysis of the *nodC* sequences of all rhizobial strains belonging to the genera *Rhizobium*, *Sinorhizobium*, *Mesorhizobium*, and *Bradyrhizobium* found significant differences between strains from NEC and NWC, even though all strains were isolated from the same host, *A. mongholicus*. The majority of the rhizobial strains from NEC and NWC were located on different and separate branches, except for the NEC strains *M. temperatum* IMUNJ 23017, IMUNJ 23015, IMUNJ 23009, and IMUNJ 23099 mentioned above, which were cross-distributed among other mesorhizobial strains from NWC.

### 3.3 Nodulation test and conserved domain characteristics of the NodC protein

**Figure 3:**
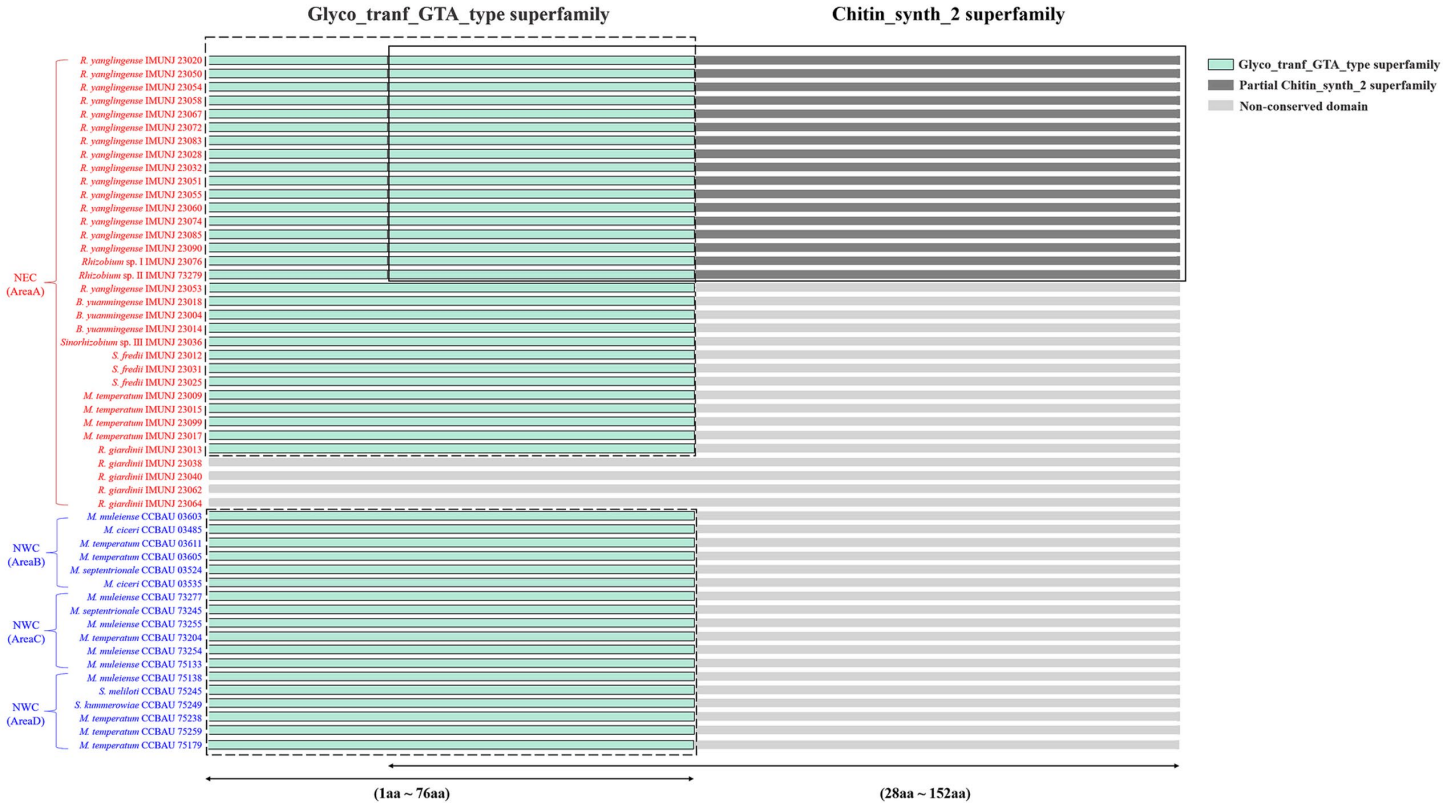
Conserved domain characteristics of NodC in rhizobial strains. Domain Glyco_tranf_GTA_type superfamily located in the 1st to 76th amino acid sequences (1aa–76aa) is colored with light green and framed with a dashed line. Domain Chitin_synth_2 superfamily located in the 28th to 152nd amino acid sequences (28aa–152aa) is colored with dark gray and framed with a solid line. The overlap of domains Glyco_tranf_GTA_type and Chitin_synth_2 superfamily located in the 28th to 76th amino acid sequences (28aa–76aa) is also colored with light green and framed with both dashed and solid lines. No conserved domain found in other amino acid sequences is colored with light gray.

All rhizobial isolates were able to reform effective nodules on the roots of *A. mongholicus*, except for four non-nodulating strains, namely *R. giardinii* IMUNJ 23040, IMUNJ 23064, IMUNJ 23038, and IMUNJ 23062. To further evaluate the effects of the gene sequence characteristics of *nodC* on the nodulation capacity, we translated the complete sequences of *nodC* into the amino acid composition of the NodC protein. Amino acid sequence analysis based on searches on the NCBI’s CDD found two conserved domains (Glyco_tranf_GTA_type superfamily and Chitin_synth_2 superfamily) in NodC (Figure 3). Moreover, we detected the conserved domain Glyco_tranf_GTA_type superfamily (Glycosyltransferase family A, GT-A)^3^ only in the NodC protein sequences of the nodulating strains; i.e., this conserved domain was not detected in four non-nodulating strains. Therefore, the Glyco_tranf_GTA_type superfamily is the most highly conserved and essential domain shared by almost all chitin synthase (CHS) proteins of rhizobial strains associated with *A. mongholicus*. Meanwhile, the conserved domain Chitin_synth_2 superfamily (CHS)^4^ was only found in the NodC protein sequences of 17 *R. yanglingense* strains, although it was absent in *R. yanglingense* IMUNJ 23053.

### 3.4 Estimates of genetic differentiation and gene flow among the rhizobial populations from NEC and NWC

We calculated the average nucleotide divergence (*Dxy*) and number of migrants (*Nm*) to estimate the genetic differentiation and gene flow among groups of rhizobial strains associated with *A. mongholicus* in NEC (area A) and in NWC comprising Shanxi (area B), Gansu (area C), and Ningxia (area D) (Table 1; Supplementary Table S4). The *Dxy* values for the concatenated core genes between NEC (area A) and NWC (areas B–D) are 0.13410 (0.13466, 0.13460, and 0.13326), which are higher than the *Dxy* values among NWC (areas B–D), and the *Dxy* values for the symbiotic *nodC* gene are 0.22712 (0.22605, 0.22724, and 0.22795). In contrast, the *Nm* values for concatenated core genes between NEC (area A) and NWC (areas B–D) are 0.83 (0.72, 0.58, and 1.16), which are lower than the *Nm* values among NWC (areas B–D), and the *Dxy* values for the symbiotic *nodC* gene are 0.97 (0.68, 0.67, and 1.67).

**Table 1:** Genetic differentiation (presented as *Dxy*) and gene flow (presented as *Nm*) in representative rhizobia of *Astragalus mongholicus* Bunge from NEC (area A) and NWC (areas B–D).

*Nm*				
*Dxy*	NEC (A)	NWC (B)	NWC (C)	NWC (D)
Concatenated core genes				
NEC (A)		0.72^***^	0.58^***^	1.16^***^
NWC (B)	0.13466^***^		14.73^ns^	7.27^ns^
NWC (C)	0.13460^***^	0.04973^ns^		9.57^ns^
NWC (D)	0.13326^***^	0.07763^ns^	0.06862^ns^	
Nodulation genes *nodC*				
NEC (A)		0.68^***^	0.67^***^	1.67^***^
NWC (B)	0.22605^***^		9.03^ns^	3.79^ns^
NWC (C)	0.22724^***^	0.02070^ns^		4.13^ns^
NWC (D)	0.22795^***^	0.07398^ns^	0.07236^ns^	

Number of migrants (*Nm*) and average nucleotide divergence between groups (*Dxy*) are shown in the upper and lower triangles of the table. ^***^p < 0.01, ^*^p < 0.05, and ^ns^*p* > 0.05.

We estimated the gene flow and genetic exchange among these rhizobial strains distributed in NEC and NWC by constructing two network trees using the Neighbor-Net algorithm based on the concatenated core genes (Supplementary Figure S1) and the symbiotic *nodC* gene (Supplementary Figure S2). Rhizobial strains isolated from NEC and NWC were located on different branches of the network trees, indicating that gene flow occurred infrequently among these rhizobial isolates from different geographic locations for both concatenated core and symbiotic *nodC* genes. Exceptions were IMUNJ 23009, IMUNJ 23015, IMUNJ 23017, and IMUNJ 23099, which were cross-distributed together with other strains in NWC such as *M. temperatum* CCBAU 73204.

### 3.5 Nucleotide diversity inferred from different genes

Although the rhizobial NWC strains were from three different areas (B–D), the nucleotide diversity (*π*) values for the concatenated core (0.06727) and symbiotic *nodC* (0.05925) genes were relatively low, while their *πN*/*πS* values were high (1.09206 and 1.56343 for the concatenated core and symbiotic *nodC* genes, respectively), as shown in Supplementary Table S3. In contrast, the *π* values of the rhizobial strains from area A in NEC are significantly high for the concatenated core (0.10000) and symbiotic *nodC* (0.24096) genes, while their *πN*/*πS* values are low (0.71056 and 1.19163 for the concatenated core and symbiotic *nodC* genes, respectively).

### 3.6 Recombination of rhizobial lineages in the evolutionary history

The values for the minimal recombination events (*Rm*) during the evolution of the core and symbiotic *nodC* genes of rhizobial strains associated with *A. mongholicus* are 117 and 41, respectively, as determined by DnaSP (Table 2). Based on our calculations, the *r*/*m* and *ρ*/*θ* values for the core genes in NEC are 7.451 and 0.302, respectively, which are lower than those for NWC (*r*/*m* = 38.721, *ρ*/*θ* = 4.117).

**Table 2:** Recombination analysis of rhizobial isolates associated with *Astragalus mongholicus* using DnaSP and CLONALFRAME software.

Genes	Length (bp)	*Rm*	*r*/*m*	*ρ*/*θ*
Concatenated core genes	1,368	117	1.502	0.139
NEC	1,368	87	7.451	0.302
NWC	1,368	63	38.721	4.117
Nodulation gene *nodC*	429	41	0.871	0.014
NEC	429	8	0.331	0.005
NWC	429	37	0.275	0.002

*Rm, minimum number of recombination events; r/m, the relative impact of recombination compared with that of mutation in the genetic diversification of the lineage; ρ/θ, the relative occurrence frequency of recombination compared with that of mutation in the history.*

**Figure 4:**
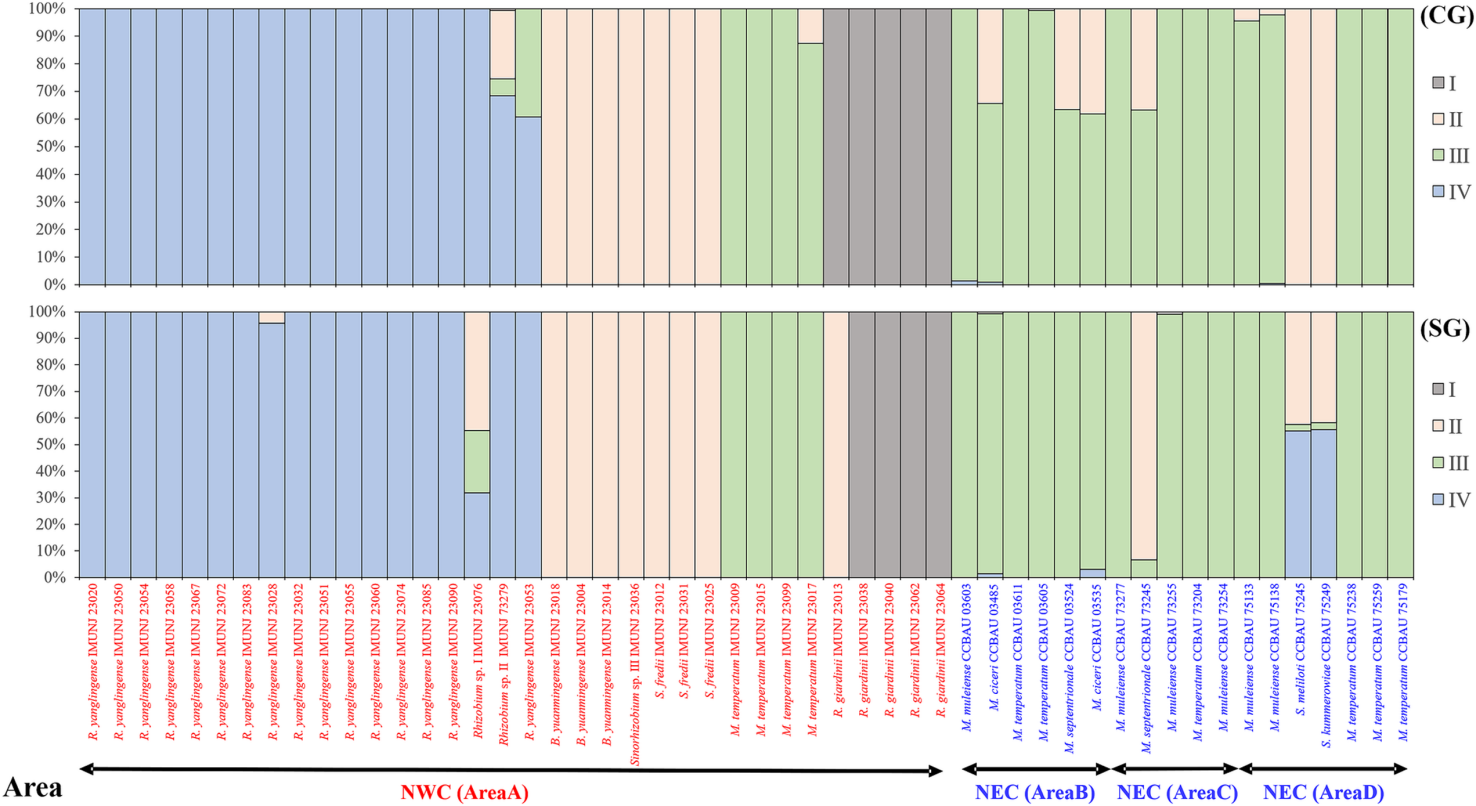
STRUCTURE analyses of rhizobial populations associated with *A. mongholicus* from NEC (area A) and NWC (areas B–D). Concatenated core gene (a) and symbiotic gene *nodC* (b) were analyzed. The inferred ancestries are designated sequentially as I, II, III, and IV shown in bars and filled with different grayness or dots. The horizontal axis represents current rhizobial individuals (from areas A to D in order), and the bar for each individual was filled according to the inferred proportions of single-nucleotide alleles that were derived from each of the ancestral lineages. The vertical axis represents the percentage of the ancestral lineages.

In STRUCTURE analyses, an optimum *K*-value of 4 was chosen according to the maximum log-likelihood number (LnP(D)) calculated with Admixture-LOCPRIOR model, clearly identifying four ancestral lineages (I–IV) as shown in Figure 4. The structural patterns of the concatenated core and symbiotic *nodC* genes are generally similar. Rhizobial strains isolated from NEC evolved from ancestral lineage IV without lineage fusion, suggesting that these strains evolved with genetic materials derived from one ancestral lineage and without gene flow. However, the genetic materials of some strains, such as IMUNJ 23017, IMUNJ 23013, IMUNJ 23053, IMUNJ 23076, and IMUNJ 23079 from NEC and CCBAU 03485, CCBAU 03524, CCBAU 03535, CCBAU 75245, and CCBAU 75249 from NWC, were derived from more than two ancestral lineages, indicating frequent gene flow during the evolutionary process.

## 4 Discussion

*Astragalus*, a primary source of traditional Chinese medicines, forms a mutualistic symbiosis with rhizobia that provides the plant with nitrogen for its growth and metabolism (Zhiyong et al., 2024). Heavy application of chemical fertilizer during cultivation affects the quality of *Astragalus* products. The selection of rhizobial strains that are capable of fixing nitrogen efficiently and adapting to the local soil environment is particularly important to improve the quality of *Astragalus* (Ji et al., 2017). However, in the absence of host plants, the majority of the free-living rhizobial strains in the soil gradually lose their ability to efficiently fix nitrogen (Ji et al., 2020). Therefore, studying the genetic differentiation and gene flow of rhizobial strains associated with *Astragalus* can provide a clearer understanding of the evolutionary process underlying the genetic diversity of plant-associated rhizobia. The symbiotic bacteria isolated from 14 commonly known *Astragalus* species have been identified as nine *Mesorhizobium* species (*M. alhagi*, *M. septentrionale*, *M. amorphae*, *M. temperatum*, *M. tianshanense*, *M. ciceri*, *M. muleiense*, *M. huakuii*, and *M. qingshengii*), two *Sinorhizobium* species (*S. meliloti* and *S. fredii*), eight *Rhizobium* species (*R. mongolense*, *R. loessense*, *R. gallicum*, *R. huautlense*, *R. tropici*, *R. galegae*, *R. leguminosarum*, and *R. giardinii*), two *Bradyrhizobium* species (*B. elkanii* and *B. japonicum*), and one non-nodulating *Agrobacterium* sp. (Wei et al., 2008; Zhao et al., 2008; Li et al., 2009; Zheng et al., 2013; Chen et al., 2015; Yan et al., 2016; Zhang et al., 2020b). However, none of the *Astragalus* species can establish a symbiotic relationship with all four genera of Rhizobacteria (*Rhizobium*, *Mesorhizobium*, *Bradyrhizobium*, and *Sinorhizobium*). The genotypes of plant-associated rhizobial strains depend mainly on their host *Astragalus* species and environmental conditions (Burghardt et al., 2022). For instance, *M. septentrionale* is present in most nodules, but especially in *Astragalus* growing on barren soils; meanwhile, *M. temperatum* is the predominant species in the nodules of *Astragalus* growing in nitrogen-rich fields (Yan et al., 2016).

*A. mongholicus* that is mainly distributed in NEC and NWC is one of the most important *Astragalus* species (Wang et al., 2023; Li et al., 2024). A systematic study of the genetic diversity and distribution of rhizobia associated with *A. mongholicus* in agricultural soils from NWC found that *M. temperatum* and *M. muleiense* are the predominant groups in this region (Yan et al., 2016). In NEC, however, *R. yanglingense* was the most predominant microsymbiont in the nodulated roots of *A. mongholicus* (Figure 1). Surprisingly, *R. yanglingense*, *R. giardinii*, *S. fredii*, and *B. yuanmingense* have never been reported to be present in the nodules of *A. mongholicus* in NWC (Yan et al., 2016). Moreover, the phylogenetic relationships among the rhizobial strains in NEC differed from those in NWC (Figure 1). *A. mongholicus* established symbiotic relationships with *M. temperatum*, *M. muleiense*, *M. septentrionale*, *M. ciceri*, *S. kummerowiae*, and *S. meliloti* in NWC. However, in NEC, it was associated with *R. yanglingense*, *B. yuanmingense*, *S. fredii*, and *M. temperatum*, where we observed an unexpectedly higher level of genetic diversity compared to the rhizobial isolates in NWC. An alkaline soil (pH 8.32) in NEC was detected and slightly higher than the mean pH value (pH 8.08) in NWC (Yan et al., 2016). However, organic carbon (OC), available potassium (AK), and available phosphorus (AP) of soil in NEC were lower than the mean values of corresponding OC (13.28 g/kg), AK (160.46 mg/kg), and AP (25.86 mg/kg) in NWC (Yan et al., 2016). The identities of the rhizobial strains isolated from *A. mongholicus* in NEC and NWC demonstrate that the distribution and community composition of rhizobia associated with this plant are dependent on the geographical location and its associated environmental conditions (Kim et al., 2022).

We were unable to detect the gene flow between rhizobial strains in NEC and NWC, as evidenced by the high *Dxy* values and low *Nm* values (Table 1; Supplementary Table S4). Our analysis indicated that the genetic materials of the rhizobial strains in NEC are derived from the ancestral lineage IV without lineage fusion (Figure 4). Moreover, no gene flow occurred throughout the strains’ evolutionary history. Our results confirmed that plant-associated rhizobial strains have evolved independently from strains that have adapted to a free-living lifestyle without intraregional recombination (Tao et al., 2021). Although these strains have been isolated from a common host, *A. mongholicus*, ecogeographical isolation due to the distance between NEC and NWC has restricted genetic drift and gene flow, which are major evolutionary forces underlying their distribution and community composition (Van Cauwenberghe et al., 2014; Liu et al., 2023). In addition, the high level of genetic diversity among rhizobial strains in NEC was due to the intraregional genetic differentiation rather than inter-regional genetic differentiation (Supplementary Figures S1, S2). Such diversity facilitated adaptation to the multiple factors in the local soil environment, such as nitrogen availability, rhizobial density, community complexity, and legume genotype (Burghardt et al., 2022). Finally, lower *r*/*m* and *ρ*/*θ* values (Table 2) suggested that the high genetic diversity of rhizobial strains in NEC was independent of recombination frequency, although the impact of recombination on chromosomal differentiation has been considerable among the different ecoregions of China, where it serves as the driver for mixing rhizobial populations (Tian et al., 2010; Van Cauwenberghe et al., 2014). Our results indicated that intraregional genetic differentiation by mutation, rather than recombination, is the main driver of high genetic diversity in NEC.

The *nodC* gene encodes N-acetylglucosaminyltransferase, which is present in all *Rhizobium* species and is required for the synthesis of the core structure of Nod factors (NFs). Moreover, the specific gene *nodC* is involved in diverse NF substitutions that confer plant specificity (Roche et al., 1996). The phylogenetic analysis classified the rhizobial strains isolated from *A. mongholicus* in NEC into five branches of the *nodC* phylogenetic tree (Figure 2). The tree branches generally corresponded to their genus and/or species definition (Figure 1). Overall, our results suggested that *A. mongholicus* is an extremely promiscuous legume without strict selectivity on either the symbiotic *nodC* gene or the species-determining core genes of rhizobia (Jiao et al., 2015). However, we also observed several cases where strains from different species or genera shared identical or highly similar *nodC* genes (Figure 2). This suggests that symbiotic genes were likely acquired through horizontal gene transfer among diverse rhizobial species. Frequent gene transfer within plant-associated microbial communities appears to facilitate the acquisition of symbiotic genes through horizontal gene transfer events (Laguerre et al., 2001; Msaddak et al., 2023). Furthermore, symbiotic genes are often clustered on extra-chromosomal replicons (megaplasmids and chromids) (Provorov et al., 2022) that construct the structure of the high genetic diversity of rhizobial strains in NEC. The higher nucleotide diversity (*π*) of *nodC* (Supplementary Table S3) was influenced by host-related natural selection and ecological selection pressures (Karasev et al., 2023). In the present study, the distribution of rhizobial species was influenced by the environmental conditions of the sampling sites in NEC and NWC, emphasizing the need to identify effective nitrogen-fixing strains for specific locations (Aserse et al., 2024).

We assumed that the characteristics of the conserved domains in NodC of rhizobial strains are related to *A. mongholicus* specificity. The conserved domain (Glyco_tranf_GTA_type superfamily) appears to be the most highly conserved and essential domain shared by all rhizobial strains that can form nodules on the roots of *A. mongholicus* (Figure 3). Such conservation suggests that the biosynthetic step of transferring a sugar moiety from an activated nucleotide-sugar donor to an acceptor molecule is necessary for various biological processes (e.g., biosynthesis of oligosaccharides, polysaccharides, and glycoconjugates) of these rhizobial strains (Wang et al., 2015). In contrast, the conserved domain (Chitin_synth_2 superfamily) was found only in *R. yanglingense* (Figure 3), the predominant species in NEC, indicating that chitin biosynthesis (Chen et al., 2022) is not essential for the function of NodC of these rhizobial strains during their symbiosis with the host *A. mongholicus*.

## 5 Conclusion

In this study, we found that rhizobial strains associated with *A. mongholicus* in NEC are highly genetically diverse and consist of at least four genera. We identified *Rhizobium yanglingense* as the predominant microsymbiont. In addition, the microsymbiont genospecies nodulating *A. mongholicus* in NEC were significantly different from those in NWC. Furthermore, the evolutionary trajectory of these rhizobial strains is consistent with intraregional genetic differentiation rather than inter-regional gene flow and recombination. Ecogeographical isolation-by-distance severely restricted genetic drift and gene flow. However, we also found inter-regional symbiont genospecies, which may be the result of similar selection by hosts rather than coevolution. Finally, the Glyco_tranf_GTA_type superfamily (Glycosyltransferase family A) is the most highly conserved and essential domain in the NodC of rhizobial strains associated with *A. mongholicus*.

## Data Availability

The original contributions presented in the study are publicly available. This data can be found here: https://www.ncbi.nlm.nih.gov, accession numbers PQ247539–PQ247572; PQ247471–PQ247504; PQ279584–PQ279617.

## Footnotes

^1^ www.worldfloraonline.org

^2^ https://www.ncbi.nlm.nih.gov/Structure/bwrpsb/bwrpsb.cgi

^3^ https://www.ncbi.nlm.nih.gov/Structure/cdd/cl11394

^4^ https://www.ncbi.nlm.nih.gov/Structure/cdd/cl37687
