# Supplementary material for: Great diverse rhizobial community nodulating Astragalus mongholicus in the northeastern region of China
Source: Front Microbiol. 2024 Dec 13;15:1507637. doi: 10.3389/fmicb.2024.1507637 (PMC11671508; doi:10.3389/fmicb.2024.1507637)
Supplement: Supplementary file 1 [file Data_Sheet_1.PDF]

Great diverse rhizobial community nodulating *Astragalus mongholicus* in the northeastern region of China

Supplementary materials

Supplementary Figure S1. Neighbor-Nets tree are constructed based upon concatenated core genes (*recA*, *atpD*, *glnII*).

Supplementary Figure S2. Neighbor-Nets tree are constructed based upon symbiotic gene *nodC*.

Supplementary Table S1. Representative rhizobia of *Astragalus mongolicus* Bunge from NEC (34 strains with red color) and NWC (18 strains with blue color) for this study.

Supplementary Table S2. Primers for these genes and annealing temperature of them (Ta, °C) in PCR amplification.

Supplementary Table S3. Nucleotide polymorphism of representative rhizobia of *Astragalus mongolicus* Bunge from NEC and NWC.

Supplementary Table S4. Genetic differentiation (presented as *Dxy*) and gene flow (presented as *Nm*) in representative rhizobia of *Astragalus mongolicus* Bunge from NEC and NWC.

Supplementary Table S5. Accession numbers of the genes obtained in NEC (34 strains with red color) and NWC (18 strains with blue color) and deposited in GenBank.

Supplementary Figure S1. Neighbor-Nets tree are constructed based upon concatenated core genes (*recA, atpD, glnII*). These representative rhizobia of *Astragalus mongolicus* Bunge, with IMUNJ before the numbers isolated from NEC and CCBAU before the numbers isolated from NWC, were highlighted with red and blue colours, respectively. The length of *recA*, *atpD* and *glnII* sequences are 440, 425 and 503 bp, respectively.

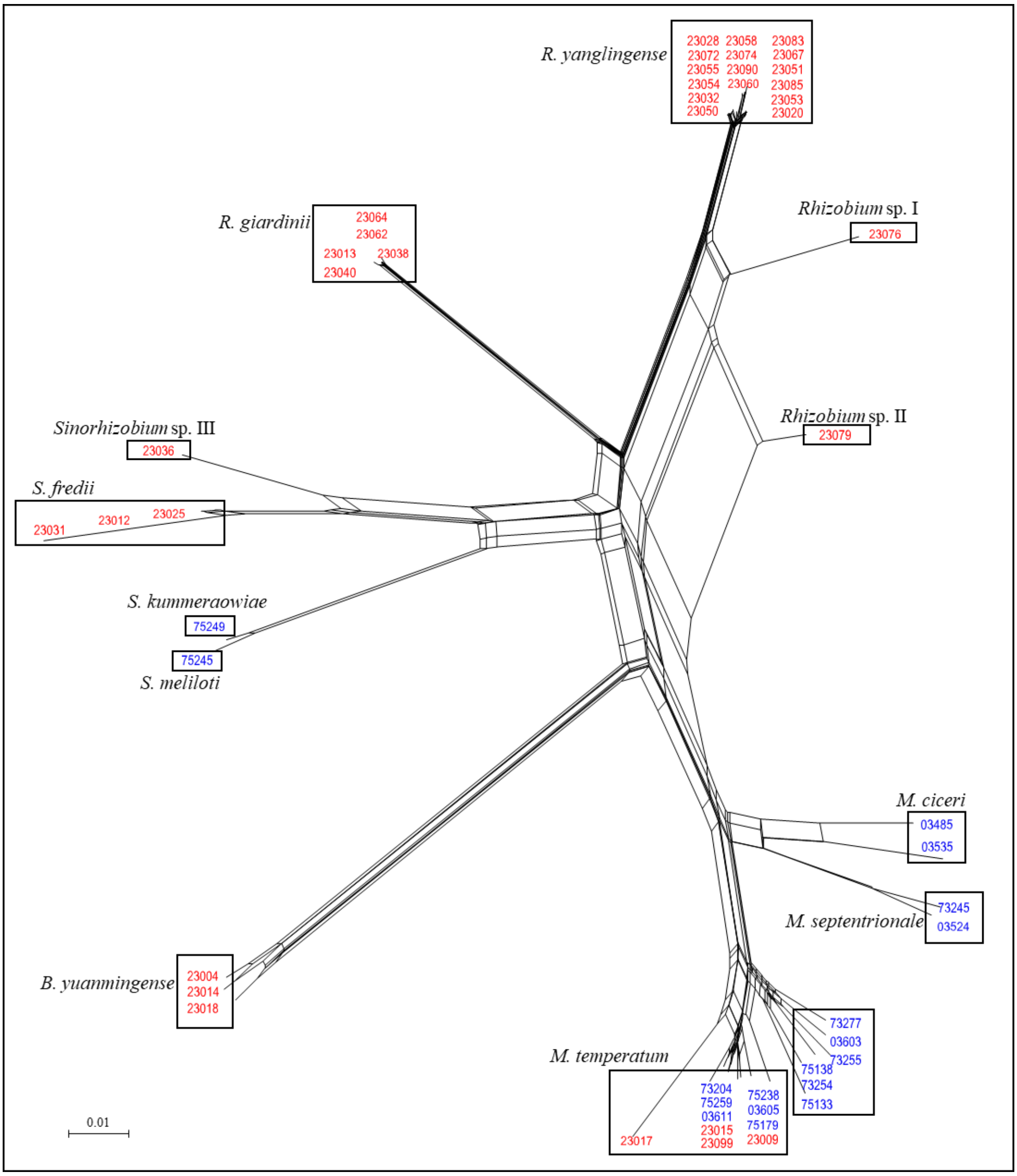

Supplementary Figure S2. Neighbor-Nets tree are constructed based upon symbiotic gene *nodC*. These representative rhizobia of *Astragalus mongolicus* Bunge, with IMUNJ before the numbers isolated from NEC and CCBAU before the numbers isolated from NWC, were highlighted with red and blue colours, respectively. The length of *nodC* sequence is 429 bp.

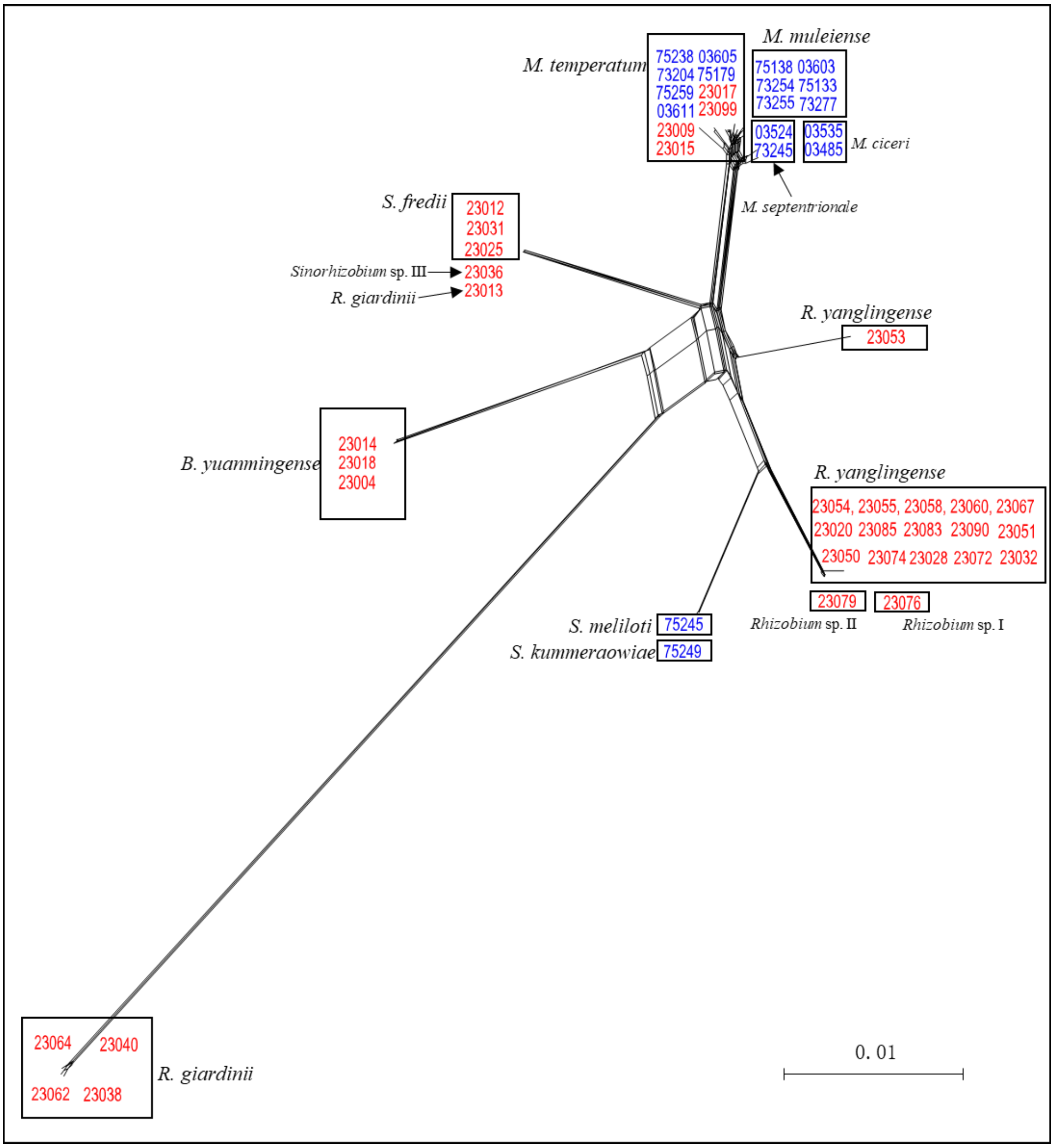

Supplementary Table S1. Representative rhizobia of *Astragalus mongolicus* Bunge from NEC (34 strains with red color) and NWC (18 strains with blue color) for this study.

Strains (No.)	Species	Areas	Collectors	Provinces	Host plants	Longitude	Latitude	
IMUNJ 23004	*B. yuanmingense*	NEC(AreaA)	Zhaojun Ji	Inner Mongolia	*A. mongholicus*	120°45′35″	42°34′45″	This study
IMUNJ 23009	*M. temperatum*	NEC(AreaA)	Zhaojun Ji	Inner Mongolia	*A. mongholicus*	120°45′35″	42°34′45″	This study
IMUNJ 23012	*S. fredii*	NEC(AreaA)	Zhaojun Ji	Inner Mongolia	*A. mongholicus*	120°45′35″	42°34′45″	This study
IMUNJ 23013	*R. giardinii*	NEC(AreaA)	Zhaojun Ji	Inner Mongolia	*A. mongholicus*	120°45′35″	42°34′45″	This study
IMUNJ 23014	*B. yuanmingense*	NEC(AreaA)	Zhaojun Ji	Inner Mongolia	*A. mongholicus*	120°45′35″	42°34′45″	This study
IMUNJ 23015	*M. temperatum*	NEC(AreaA)	Zhaojun Ji	Inner Mongolia	*A. mongholicus*	120°45′35″	42°34′45″	This study
IMUNJ 23017	*M. temperatum*	NEC(AreaA)	Zhaojun Ji	Inner Mongolia	*A. mongholicus*	120°45′35″	42°34′45″	This study
IMUNJ 23018	*B. yuanmingense*	NEC(AreaA)	Zhaojun Ji	Inner Mongolia	*A. mongholicus*	120°45′35″	42°34′45″	This study
IMUNJ 23020	*R. yanglingense*	NEC(AreaA)	Zhaojun Ji	Inner Mongolia	*A. mongholicus*	120°45′35″	42°34′45″	This study
IMUNJ 23025	*S. fredii*	NEC(AreaA)	Zhaojun Ji	Inner Mongolia	*A. mongholicus*	120°45′35″	42°34′45″	This study
IMUNJ 23028	*R. yanglingense*	NEC(AreaA)	Zhaojun Ji	Inner Mongolia	*A. mongholicus*	120°45′35″	42°34′45″	This study
IMUNJ 23031	*S. fredii*	NEC(AreaA)	Zhaojun Ji	Inner Mongolia	*A. mongholicus*	120°45′35″	42°34′45″	This study
IMUNJ 23032	*R. yanglingense*	NEC(AreaA)	Zhaojun Ji	Inner Mongolia	*A. mongholicus*	120°45′35″	42°34′45″	This study
IMUNJ 23036	*Sinorhizobium* sp. III	NEC(AreaA)	Zhaojun Ji	Inner Mongolia	*A. mongholicus*	120°45′35″	42°34′45″	This study
IMUNJ 23038	*R. giardinii*	NEC(AreaA)	Zhaojun Ji	Inner Mongolia	*A. mongholicus*	120°45′35″	42°34′45″	This study
IMUNJ 23040	*R. giardinii*	NEC(AreaA)	Zhaojun Ji	Inner Mongolia	*A. mongholicus*	120°45′35″	42°34′45″	This study
IMUNJ 23050	*R. yanglingense*	NEC(AreaA)	Zhaojun Ji	Inner Mongolia	*A. mongholicus*	120°45′35″	42°34′45″	This study
IMUNJ 23051	*R. yanglingense*	NEC(AreaA)	Zhaojun Ji	Inner Mongolia	*A. mongholicus*	120°45′35″	42°34′45″	This study
IMUNJ 23053	*M. temperatum*	NEC(AreaA)	Zhaojun Ji	Inner Mongolia	*A. mongholicus*	120°45′35″	42°34′45″	This study
IMUNJ 23054	*R. yanglingense*	NEC(AreaA)	Zhaojun Ji	Inner Mongolia	*A. mongholicus*	120°45′35″	42°34′45″	This study
IMUNJ 23055	*R. yanglingense*	NEC(AreaA)	Zhaojun Ji	Inner Mongolia	*A. mongholicus*	120°45′35″	42°34′45″	This study
IMUNJ 23058	*R. yanglingense*	NEC(AreaA)	Zhaojun Ji	Inner Mongolia	*A. mongholicus*	120°45′35″	42°34′45″	This study
IMUNJ 23060	*R. yanglingense*	NEC(AreaA)	Zhaojun Ji	Inner Mongolia	*A. mongholicus*	120°45′35″	42°34′45″	This study
IMUNJ 23062	*R. giardinii*	NEC(AreaA)	Zhaojun Ji	Inner Mongolia	*A. mongholicus*	120°45′35″	42°34′45″	This study
IMUNJ 23064	*R. giardinii*	NEC(AreaA)	Zhaojun Ji	Inner Mongolia	*A. mongholicus*	120°45′35″	42°34′45″	This study
IMUNJ 23067	*R. yanglingense*	NEC(AreaA)	Zhaojun Ji	Inner Mongolia	*A. mongholicus*	120°45′35″	42°34′45″	This study
IMUNJ 23072	*R. yanglingense*	NEC(AreaA)	Zhaojun Ji	Inner Mongolia	*A. mongholicus*	120°45′35″	42°34′45″	This study
IMUNJ 23074	*R. yanglingense*	NEC(AreaA)	Zhaojun Ji	Inner Mongolia	*A. mongholicus*	120°45′35″	42°34′45″	This study
IMUNJ 23076	*Rhizobium* sp. I	NEC(AreaA)	Zhaojun Ji	Inner Mongolia	*A. mongholicus*	120°45′35″	42°34′45″	This study

IMUNJ 23079	*Rhizobium* sp. II	NEC(AreaA)	Zhaojun Ji	Inner Mongolia	*A. mongholicus*	120°45′35″	42°34′45″	This study
IMUNJ 23083	*R. yanglingense*	NEC(AreaA)	Zhaojun Ji	Inner Mongolia	*A. mongholicus*	120°45′35″	42°34′45″	This study
IMUNJ 23085	*R. yanglingense*	NEC(AreaA)	Zhaojun Ji	Inner Mongolia	*A. mongholicus*	120°45′35″	42°34′45″	This study
IMUNJ 23090	*R. yanglingense*	NEC(AreaA)	Zhaojun Ji	Inner Mongolia	*A. mongholicus*	120°45′35″	42°34′45″	This study
IMUNJ 23099	*M. temperatum*	NEC(AreaA)	Zhaojun Ji	Inner Mongolia	*A. mongholicus*	120°45′35″	42°34′45″	This study
CCBAU 03485	*M. ciceri*	NWC(AreaB)	Hui Yan	Shanxi	*A. mongholicus*	113°45′30.462″	39°25′57.585″	Previous (Yan et al., 2016)
CCBAU 03524	*M. septentrionale*	NWC(AreaB)	Hui Yan	Shanxi	*A. mongholicus*	113°45′30.462″	39°25′57.585″	Previous (Yan et al., 2016)
CCBAU 03535	*M. ciceri*	NWC(AreaB)	Hui Yan	Shanxi	*A. mongholicus*	113°45′30.462″	39°25′57.585″	Previous (Yan et al., 2016)
CCBAU 03603	*M. muleiense*	NWC(AreaB)	Hui Yan	Shanxi	*A. mongholicus*	112°36′58.271″	36°59′59.582″	Previous (Yan et al., 2016)
CCBAU 03605	*M. temperatum*	NWC(AreaB)	Hui Yan	Shanxi	*A. mongholicus*	112°36′58.271″	36°59′59.582″	Previous (Yan et al., 2016)
CCBAU 03611	*M. temperatum*	NWC(AreaB)	Hui Yan	Shanxi	*A. mongholicus*	112°36′58.271″	36°59′59.582″	Previous (Yan et al., 2016)
CCBAU 73204	*M. temperatum*	NWC(AreaC)	Hui Yan	Gansu	*A. mongholicus*	104°24′42.556″	35°04′36.277″	Previous (Yan et al., 2016)
CCBAU 73245	*M. septentrionale*	NWC(AreaC)	Hui Yan	Gansu	*A. mongholicus*	104°03′54.723	34°32′45.599″	Previous (Yan et al., 2016)
CCBAU 73254	*M. muleiense*	NWC(AreaC)	Hui Yan	Gansu	*A. mongholicus*	104°03′54.723	34°32′45.599	Previous (Yan et al., 2016)
CCBAU 73255	*M. muleiense*	NWC(AreaC)	Hui Yan	Gansu	*A. mongholicus*	104°03′54.723″	34°32′45.599	Previous (Yan et al., 2016)
CCBAU 73277	*M. muleiense*	NWC(AreaC)	Hui Yan	Gansu	*A. mongholicus*	104°03′22.441″	34°24′14.061″	Previous (Yan et al., 2016)
CCBAU 75133	*M. muleiense*	NWC(AreaD)	Hui Yan	Ningxia	*A. mongholicus*	105°51′44″	35°33′37″	Previous (Yan et al., 2016)
CCBAU 75138	*M. muleiense*	NWC(AreaD)	Hui Yan	Ningxia	*A. mongholicus*	105°51′44″	35°33′37″	Previous (Yan et al., 2016)
CCBAU 75179	*M. temperatum*	NWC(AreaD)	Hui Yan	Ningxia	*A. mongholicus*	105°58′04″	35°34′57″	Previous (Yan et al., 2016)
CCBAU 75238	*M. temperatum*	NWC(AreaD)	Hui Yan	Ningxia	*A. mongholicus*	106°15′09.129″	37°46′59.535″	Previous (Yan et al., 2016)
CCBAU 75245	*S. meliloti*	NWC(AreaD)	Hui Yan	Ningxia	*A. mongholicus*	106°15′09.129″	37°46′59.535″	Previous (Yan et al., 2016)
CCBAU 75249	*S. kummerowiae*	NWC(AreaD)	Hui Yan	Ningxia	*A. mongholicus*	106°15′09.129″	37°46′59.535″	Previous (Yan et al., 2016)
CCBAU 75259	*M. temperatum*	NWC(AreaD)	Hui Yan	Ningxia	*A. mongholicus*	106°15′09.129″	37°46′59.535″	Previous (Yan et al., 2016)

Supplementary Table S2. Primers for these genes and annealing temperature of them (Ta, °C) in PCR amplification.

**Genes**	**Complete name or function**	**Preferred primers (5'→3', forward/reverse)**	**Ta (°C)**
atpD	F_0F_1 ATP synthase subunit beta	GCTSGGCCGCATCMTSAACGTC/ GCCGACACTTCMGAACCNGCCTG	58
glnII	Glutamine synthetase II protein	YAAGCTCGAGTACATYTGGCT/ TGCATGCCSGAGCCGTTCCCA	58
recA	Recombinase A	TTCGGCAAGGGMTCGRTSATG/ ACATSACRCCGATCTTCATGC	54
nodC	N-acetylglucosaminyltransferase, NodC	TGATYGAYATGGARTAYTGGCT/ CGYGACARCCARTCGCTRTTG	52

Supplementary Table S3. Nucleotide polymorphism of representative rhizobia of *Astragalus mongolicus* Bunge from NEC and NWC.

**Site (Strain No.)**	**Length (bp)**	***S***	***Eta***	***h/Hd***	π	π_S	π_N	π_N/π_S
**Concatenated core genes**								
NEC (34)	1375	482	582	23/0.963	0.10000	0.12711	0.09032	0.71056
NWC (18)	1375	332	382	18/1.000	0.06727	0.08744	0.09549	1.09206
**Nodulation gene *nodC***								
NEC (34)	429	318	420	15/0.793	0.24096	0.21447	0.25557	1.19163
NWC (18)	429	114	126	14/0.954	0.05925	0.04146	0.06482	1.56343

Note: *S*, segregating sites or number of polymorphic (segregating) sites; *Eta*, total number of mutations. *h*, haplotype number; *Hd*, haplotype diversity; π, average number of nucleotide differences per site between two sequences; π_S, nucleotide diversity for synonymous substitutions; π_N, nucleotide diversity for nonsynonymous substitutions.

Supplementary Table S4. Genetic differentiation (presented as *Dxy*) and gene flow (presented as *Nm*) in representative rhizobia of *Astragalus mongolicus* Bunge from NEC and NWC.

Nm / *Dxy*	NWC	NEC
**Concatenated core genes**		
NEC		0.83***
NWC	0.13410***	
**Nodulation genes *nodC***		
NEC		0.97***
NWC	0.22712***	

Note: Number of migrants (*Nm*) and average nucleotide divergence between groups (*Dxy*) are shown in the upper and lower triangular of the table. ***, *P*<0.01, *, *P*<0.05, ns, *P*>0.05.

Supplementary Table S5. Accession numbers of the genes obtained in NEC (34 strains with red color) and NWC (18 strains with blue color) and type strains (14 strains with black color) and deposited in GenBank.

NO.	**strain**	***recA* accession**	***atpD* accession**	***glnII* accession**	***nodC* accession**
1	IMUNJ 23004	PQ247471	PQ247505	PQ247539	PQ279588
2	IMUNJ 23009	PQ247472	PQ247506	PQ247540	PQ279589
3	IMUNJ 23012	PQ247473	PQ247507	PQ247541	PQ279590
4	IMUNJ 23013	PQ247474	PQ247508	PQ247542	PQ279591
5	IMUNJ 23014	PQ247475	PQ247509	PQ247543	PQ279592
6	IMUNJ 23015	PQ247476	PQ247510	PQ247544	PQ279593
7	IMUNJ 23017	PQ247477	PQ247511	PQ247545	PQ279594
8	IMUNJ 23018	PQ247478	PQ247512	PQ247546	PQ279595
9	IMUNJ 23020	PQ247479	PQ247513	PQ247547	PQ279596
10	IMUNJ 23025	PQ247480	PQ247514	PQ247548	PQ279597
11	IMUNJ 23028	PQ247481	PQ247515	PQ247549	PQ279598
12	IMUNJ 23031	PQ247482	PQ247516	PQ247550	PQ279599
13	IMUNJ 23032	PQ247483	PQ247517	PQ247551	PQ279600
14	IMUNJ 23036	PQ247484	PQ247518	PQ247552	PQ279601
15	IMUNJ 23038	PQ247485	PQ247519	PQ247553	PQ279584
16	IMUNJ 23040	PQ247486	PQ247520	PQ247554	PQ279585
17	IMUNJ 23050	PQ247487	PQ247521	PQ247555	PQ279602
18	IMUNJ 23051	PQ247488	PQ247522	PQ247556	PQ279603
19	IMUNJ 23053	PQ247489	PQ247523	PQ247557	PQ279604
20	IMUNJ 23054	PQ247490	PQ247524	PQ247558	PQ279605
21	IMUNJ 23055	PQ247491	PQ247525	PQ247559	PQ279606
22	IMUNJ 23058	PQ247492	PQ247526	PQ247560	PQ279607
23	IMUNJ 23060	PQ247493	PQ247527	PQ247561	PQ279608
24	IMUNJ 23062	PQ247494	PQ247528	PQ247562	PQ279586
25	IMUNJ 23064	PQ247495	PQ247529	PQ247563	PQ279587
26	IMUNJ 23067	PQ247496	PQ247530	PQ247564	PQ279609
27	IMUNJ 23072	PQ247497	PQ247531	PQ247565	PQ279610
28	IMUNJ 23074	PQ247498	PQ247532	PQ247566	PQ279611
29	IMUNJ 23076	PQ247499	PQ247533	PQ247567	PQ279612
30	IMUNJ 23079	PQ247500	PQ247534	PQ247568	PQ279613
31	IMUNJ 23083	PQ247501	PQ247535	PQ247569	PQ279614
32	IMUNJ 23085	PQ247502	PQ247536	PQ247570	PQ279615
33	IMUNJ 23090	PQ247503	PQ247537	PQ247571	PQ279616
34	IMUNJ 23099	PQ247504	PQ247538	PQ247572	PQ279617
35	CCBAU 03485	KJ556389	KJ556432	KJ556475	KJ729181
36	CCBAU 03524	KJ556392	KJ556435	KJ680396	KJ729183
37	CCBAU 03535	KJ556393	KJ556436	KJ556478	KJ729184
38	CCBAU 03603	KJ556395	KJ556438	KJ556480	KJ729185
39	CCBAU 03605	KJ556396	KJ556439	KJ556481	KJ729186
40	CCBAU 03611	KJ556397	KJ556440	KJ556482	KJ729187
41	CCBAU 73204	KJ556400	KJ556443	KJ556485	KJ729190
42	CCBAU 73245	KJ556402	KJ556445	KJ556487	KJ729192
43	CCBAU 73254	KJ556404	KJ556447	KJ556489	KJ729194
44	CCBAU 73255	KJ556405	KJ556448	KJ556490	KJ729195
45	CCBAU 73277	KJ556406	KJ556449	KJ556491	KJ729196
46	CCBAU 75133	KJ556414	KJ556457	KJ556499	KJ729204
47	CCBAU 75138	KJ556415	KJ556458	KJ556500	KJ729205
48	CCBAU 75179	KJ556418	KJ556461	KJ556503	KJ729208
49	CCBAU 75238	KJ953902	KJ953897	KJ953898	KJ953901
50	CCBAU 75245	KJ556428	KJ556471	KJ556512	KM373710

51	CCBAU 75249	KJ556430	KJ556473	KJ556514	KM373711
52	CCBAU 75259	KJ556424	KJ556467	KJ556509	KJ729214
53	**SH22623^{T}**	AY907359	AY907373	AY929462	ns
54	**USDA 1844^{T}**	AY907358	AY907372	AY929453	GQ507367
55	**MSDJ1109^{T}**	AB253203	HM142762	EU488785	ns
56	**USDA 9039^{T}**	AJ 294372	AJ 294396	AF169583	ns
57	**H152^{T}**	HQ394251	HQ394216	EU488778	AF217267
58	**CCBAU 10071^{T}**	AY591566	AY386760	AY386780	ns
59	**SDW014^{T}**	EF639843	DQ345070	EU249387	GQ167237
60	**USDA 3383^{T}**	AJ 294367	AJ 294395	AF169580	ns
61	**LMG 17148^{T}**	AM182157	AM418768	AF169578	ns
62	**CCBAU 83963^{T}**	HQ316782	HQ316724	HQ316739	HQ316752
63	**SDW018^{T}**	EF639844	D0659499	DO345073	EU130395
64	**CCBAU 71714^{T}**	AB253216	GU994044	GU994062	GU994071
65	**USDA 1002^{T}**	AJ 294382	AJ 294400	DQ767676	ns
66	**USDA 205^{T}**	AJ 294379	AJ 294402	AF169591	GU994072

Note: ns means that the *nodC* accession number has not been deposited in GenBank.
